# Key lifestyles and interim health outcomes for effective interventions in general populations: A network analysis of a large international observational study

**DOI:** 10.7189/jogh.13.04125

**Published:** 2023-10-20

**Authors:** Jiaying Li, Daniel Yee Tak Fong, Kris Yuet Wan Lok, Janet Yuen Ha Wong, Mandy Man Ho, Edmond Pui Hang Choi, Vinciya Pandian, Patricia M Davidson, Wenjie Duan, Marie Tarrant, Jung Jae Lee, Chia-Chin Lin, Oluwadamilare Akingbade, Khalid M Alabdulwahhab, Mohammad Shakil Ahmad, Mohamed Alboraie, Meshari A Alzahrani, Anil S Bilimale, Sawitree Boonpatcharanon, Samuel Byiringiro, Muhammad Kamil Che Hasan, Luisa Clausi Schettini, Walter Corzo, Josephine M De Leon, Anjanette S De Leon, Hiba Deek, Fabio Efficace, Mayssah A El Nayal, Fathiya El-Raey, Eduardo Ensaldo-Carrasco, Pilar Escotorin, Oluwadamilola Agnes Fadodun, Israel Opeyemi Fawole, Yong-Shian Shawn Goh, Devi Irawan, Naimah Ebrahim Khan, Binu Koirala, Ashish Krishna, Cannas Kwok, Tung Thanh Le, Daniela Giambruno Leal, Miguel Ángel Lezana-Fernández, Emery Manirambona, Leandro Cruz Mantoani, Fernando Meneses-González, Iman Elmahdi Mohamed, Madeleine Mukeshimana, Chinh Thi Minh Nguyen, Huong Thi Thanh Nguyen, Khanh Thi Nguyen, Son Truong Nguyen, Mohd Said Nurumal, Aimable Nzabonimana, Nagla Abdelrahim Mohamed Ahmed Omer, Oluwabunmi Ogungbe, Angela Chiu Yin Poon, Areli Reséndiz-Rodriguez, Busayasachee Puang-Ngern, Ceryl G Sagun, Riyaz Ahmed Shaik, Nikhil Gauri Shankar, Kathrin Sommer, Edgardo Toro, Hanh Thi Hong Tran, Elvira L Urgel, Emmanuel Uwiringiyimana, Tita Vanichbuncha, Naglaa Youssef

**Affiliations:** 1 School of Nursing, Li Ka Shing Faculty of Medicine, University of Hong Kong, Hong Kong SAR, China; 2 School of Nursing and Health Studies, Hong Kong Metropolitan University, Hong Kong SAR, China; 3 School of Nursing, Johns Hopkins University, Baltimore, Maryland, USA; 4 Vice-Chancellor and Principal, University of Wollongong, Wollongong, Australia; 5 Department of Social Work, East China University of Science and Technology, Shanghai, China; 6 School of Nursing, The University of British Columbia, Kelowna British Columbia, Canada; 7 The Nethersole School of Nursing, The Chinese University of Hong Kong, Hong Kong; 8 Institute of Nursing Research, Osogbo, Osun State, Nigeria; 9 College of Medicine, Maimaah University, Al Majmaah, Saudi Arabia; 10 Department of Family & Community Medicine, College of Medicine, Majmaah University, Majmaah, Saudi Arabia; 11 Department of Internal Medicine, Al-Azhar University, Cairo, Egypt; 12 Department of Urology, College of Medicine, Majmaah University, Al Majmaah, Saudi Arabia; 13 School of Public Health, JSS Medical College, JSS AHER, Mysuru, India; 14 Department of Statistics, Chulalongkorn Business School, Bangkok, Thailand; 15 Kulliyyah of Nursing, International Islamic University, Kuantan, Malaysia; 16 Italian Association against Leukemia, Lymphoma and Myeloma (AIL), Rome, Italy; 17 Diálogos Guatemala, Guatemala, Guatemala; 18 School of Nursing, Centro Escolar University, Manila, Philippines; 19 Nursing Department, Faculty of Health Science, Beirut Arab University, Lebanon; 20 Italian Group for Adult Hematologic Diseases (GIMEMA), Data Center and Health Outcomes Research Unit, Rome, Italy; 21 Department of Psychology, Beirut Arab University, Lebanon; 22 Department of hepatogastroenterology and infectious diseases, Damietta faculty of medicine, Al-Azher University, Egypt; 23 Ergonomics Research Center (ECR), University of Guadalajara, Jalisco, Mexico; 24 Laboratory of Applied Prosocial Research, Department of Basic, Developmental and Educational Psychology, Autonomous University of Barcelona, Spain; 25 Faculty of Health Sciences, University of Lethbridge, Alberta, Canada; 26 Faculty of Nursing, Ladoke Akintola University of Technology, Ogbomosho, Nigeria; 27 Alice Lee Centre for Nursing Studies, National University of Singapore, Singapore; 28 School of Nursing, Wijaya Husada Health Institute, Bogor, Indonesia; 29 Department of Optometry, University of Kwazulu-Natal, Durban, South Africa; 30 Ecove, Ghaziabad, India; 31 School of Nursing, Paramedicine and Health Care Science, Charles Sturt University, New South Wales, Australia; 32 Nam Dinh University of Nursing, Nam Dinh, Vietnam; 33 Pontificia Universidad Católica de Valparaíso, School of Social Work, Valparaíso, Chile; 34 Research Department, National Commission for Medical Arbitration, Mexico, Mexico; 35 College of Medicine and Health Sciences, University of Rwanda, Kigali, Rwanda; 36 Laboratory of Research in Respiratory Physiotherapy (LFIP), Department of Physiotherapy, State University of Londrina (UEL), Londrina, Brazil; 37 Pharmacology and Toxicology Department, Faculty of Pharmacy, Benghazi University, Libya; 38 School of Nursing and Midwifery, College of Medicine and Health Sciences, University of Rwanda, Kigali, Rwanda; 39 Center for Language Enhancement, College of Arts and Social Sciences, University of Rwanda, Huye, Rwanda; 40 Faculty of Medicine, Alzaiem Alazhari University, Khartoum North, Sudan; 41 Faculty of Health Sciences and Sports, Macao Polytechnic University, Macao; 42 National Autonomous University of Mexico, Mexico; 43 Mental Health and Learning division, Wrexham Maelor Hospital, Wrexham, United Kingdom; 44 Medical-surgical Nursing Department, Faculty of Nursing, Cairo University, Egypt

## Abstract

**Background:**

The interconnected nature of lifestyles and interim health outcomes implies the presence of the central lifestyle, central interim health outcome and bridge lifestyle, which are yet to be determined. Modifying these factors holds immense potential for substantial positive changes across all aspects of health and lifestyles. We aimed to identify these factors from a pool of 18 lifestyle factors and 13 interim health outcomes while investigating potential gender and occupation differences.

**Methods:**

An international cross-sectional study was conducted in 30 countries across six World Health Organization regions from July 2020 to August 2021, with 16 512 adults self-reporting changes in 18 lifestyle factors and 13 interim health outcomes since the pandemic.

**Results:**

Three networks were computed and tested. The central variables decided by the expected influence centrality were consumption of fruits and vegetables (centrality = 0.98) jointly with less sugary drinks (centrality = 0.93) in the lifestyles network; and quality of life (centrality = 1.00) co-dominant (centrality = 1.00) with less emotional distress in the interim health outcomes network. The overall amount of exercise had the highest bridge expected influence centrality in the bridge network (centrality = 0.51). No significant differences were found in the network global strength or the centrality of the aforementioned key variables within each network between males and females or health workers and non-health workers (all *P*-values >0.05 after Holm-Bonferroni correction).

**Conclusions:**

Consumption of fruits and vegetables, sugary drinks, quality of life, emotional distress, and the overall amount of exercise are key intervention components for improving overall lifestyle, overall health and overall health via lifestyle in the general population, respectively. Although modifications are needed for all aspects of lifestyle and interim health outcomes, a larger allocation of resources and more intensive interventions were recommended for these key variables to produce the most cost-effective improvements in lifestyles and health, regardless of gender or occupation.

Preventing and controlling of noncommunicable diseases has emerged as a critical priority for the 21st century. Lifestyle factors, such as poor diet, physical inactivity, tobacco and alcohol use, contribute significantly to the prevalence of noncommunicable diseases, constituting over one-third of the global burden of chronic diseases [1]. Embracing healthy lifestyles can substantially increase life expectancy, mitigate memory decline and improve life-years free of major chronic diseases [2-4]. However, given the scarcity of resources and the resource-intensive nature of lifestyle interventions, impact of lifestyle interventions on the general population remains understudied. Previous interventions have predominantly focused on individual aspects such as exercise [5-7], nutrition [5-7] or screen time [8], with only a few examining influences of multiple lifestyle factors [9,10]. It remains uncertain which specific lifestyle factor exerts the greatest influence on overall interim health outcomes. Identifying the most effective lifestyle factor holds immense potential for targeted and cost-effective interventions to alleviate the burden of noncommunicable diseases on a global scale, particularly in resource-limited settings and developing countries.

Unfortunately, existing studies have methodological limitations in achieving this goal due to the intricate correlations among various aspects of lifestyle and interim health outcomes. For instance, physical inactivity is associated with other lifestyle behaviours, such as smoking, alcohol consumption and an unhealthy diet [11]. Furthermore, physical inactivity affects various interim health outcomes, including mental health and sleep quality [12,13]. Moreover, behaviours like smoking, drinking, and unhealthy eating also impact interim health outcomes, such as mental health and sleep quality. Therefore, identifying the most influential variables within this complex network of interrelated factors using traditional methodologies, such as correlation analysis and regression analysis, is not feasible. However, this presents an opportunity for network analysis to address this research gap by identifying key variables: central lifestyle and interim health outcome variables that have substantial influence within their respective groups, as well as bridge lifestyle variables that significantly impact all interim health outcome variables in the other group [14]. These central and bridge variables hold the potential to serve as the most cost-effective intervention components, guiding researchers and policymakers in strategically allocating their resources and efforts [14]. While network analysis has experienced remarkable advancements within the field of psychopathology, its vast potential within health research remains largely untapped.

This study aimed to enhance our understanding of the complex interplay between lifestyles and interim health outcomes. We sought to identify the most influential variables among various lifestyles (central lifestyles) and multiple interim health outcome factors (central interim health outcomes) as well as the most impactful lifestyles carrying the biggest influence on multiple interim health outcomes as a whole (bridge lifestyles). Additionally, we investigated potential differences in these relationships based on gender and occupation. The findings bear significant implications for improving public health by identifying the most cost-effective intervention components and guiding more efficient allocation of limited resources.

## METHODS

### Study settings

This study investigated populations from 30 territories across six World Health Organization (WHO) regions, including Australia, Brazil, Burundi, Canada, Chile, Egypt, Guatemala, Hong Kong, India, Indonesia, Italy, Lebanon, Libya, Macau, mainland China, Malaysia, Mexico, Nigeria, the Philippines, the Republic of Sudan, Rwanda, Saudi Arabia, Singapore, South Africa, South Korea, Spain, Thailand, the United Kingdom, the United States and Vietnam. Territories were strategically selected to ensure representation from the six WHO regions and diverse economic development levels, thereby enhancing the generalisability of our findings. Participants were primarily recruited through online platforms and they voluntarily completed the survey in their preferred language. Details can be found in the published protocol [15].

### Participants and sample size

This study employed convenient sampling from 30 territories with specific eligibility criteria that required participants to be adults aged 18 or above and possess the ability to complete the questionnaire in their respective language. We recruited participants aged 18 or older from 30 countries to complete the questionnaire. With a maximum of 28 nodes and an estimated 378 edges in our network, we determined a required sample size of 1134 participants based on the guideline of at least three participants per parameter [16].

### Measures

#### Socio-demographics

The sociodemographic variables included gender, age, country, marital status, highest education attained, employment, perceived social rank and whether the participant was a practicing health professional.

#### Measuring methods of outcomes

Participants rated the change in 18 lifestyle factors and 13 interim health outcomes during the coronavirus disease 2019 (COVID-19) compared to pre-pandemic using a 5-point Likert scale (1 = substantially reduced, 5 = substantially increased, 3 = no change). Given the pandemic's context, the study focused on assessing outcome changes rather than absolute levels. This approach had promising potential for post-pandemic applicability, assuming an equal-magnitude but opposite-direction rebound effect on the assessed outcomes. Importantly, the change in direction of all variables did not affect the network and centrality of variables.

#### Lifestyles and interim health outcomes

The development of lifestyle and interim health outcome items was outlined in the published protocol [15]. Specifically, the questionnaire underwent a systematic development and translation process. Initially, an English draft was formulated based on an extensive literature review on the impact of COVID-19 on lifestyle and interim health outcomes. Insights from a multidisciplinary team of experts enriched the questionnaire, and refined for clarity. The questionnaire was then reviewed by international experts to ensure cultural appropriateness. The translation followed a rigorous forward-backward method. To enhance clarity, this translated version was tested with five native speakers. Prior to its final release, a pilot test was conducted with at least 10 native speakers from each language, ensuring both consistency and clarity across diverse regions. The lifestyle areas examined included food types in daily meals, consumption of fruits and vegetables, consumption of frozen food/food products, consumption of snacks, soft drinks/juices/other sugary drinks, having a meal at home, cooking at home, eating takeout food, taking alternative medicine or natural health products, taking oral supplements/vitamins, smoking tobacco, alcohol consumption, duration of sitting, duration of screen time, frequency of exercise, duration of exercise, type of exercise, and overall amount of exercise. interim health outcomes included weight, appetite, physical health, sleep quality, quality of life, mental burden, emotional distress, family disputes, social support provided, social support received, social activities, income and economic burden.

### Data collection

Data were collected through online survey platforms (project website: https://care.hku.hk or customised links) and offline electronic forms (including PDF format for areas with limited internet access). Additionally, participation was incentivised with the Hong Kong dollar (HK$)1 donation to the Red Cross for each completed questionnaire.

### Statistical analysis

The collected data were organised in a Microsoft Excel database and underwent thorough quality control procedures. Analysis was conducted using R Statistical Software (v4.1.1; R Core Team 2021). Descriptive statistics summarised participants' demographics and perceptions of COVID-19's impact on lifestyles and interim health outcomes. Specifically, variables were assessed for normality using P-P plots and reported as mean and standard deviation, while categorical variables were reported as frequency and percentage. Network analyses were performed across five domains, including checking topological overlap, network estimation, network stability, calculation of centrality and bridge centrality indices, and network comparison tests.

#### Rationale for choosing network analysis

To capture the complex interplay between lifestyle factors and interim health outcomes, we chose network analysis over traditional correlation analysis. While the latter efficiently gauges linear relationships between two variables, it becomes limited when confronted with the complexity of multiple interconnected relationships. In contrast, network analysis provides a more comprehensive perspective. It visualises each variable as a “node” and connects them with “edges” to depict relationships, taking account all other variables within the network. This approach enables us to examine both direct pairwise interactions and the broader relational structure, spotlighting key influencers or connectors within and between networks. Given the interwoven nature of our data set, employing network analysis was crucial in comprehending the intricate relationships and identifying key variables.

#### Checking topological overlap

We utilised the goldbricker function in the R package networktools to compare the correlations and identify unique variables, ensuring the network analysis avoided artificial relationships caused by similar symptoms. A significance proportion of 0.25 for inclusion and *P* < 0.01 were used to determine statistical significance [17].

#### Network estimation

Three networks were obtained: one comprising all lifestyles, one comprising all interim health outcomes and a bridge network linking the two. Nodes represented items in the networks and edges depicted their relationships. We employed partial correlation analysis to estimate pairwise associations while controlling for the confounding effects of all other nodes. The least absolute shrinkage and selection operator (LASSO) method was applied to shrink edges and set small correlations to zero. The extended Bayesian Information Criteria (EBIC) was used to select a related turning parameter and create a more interpretable and sparser network [16]. We used the R packages bootnet and qgraph to estimate and visualise the network, respectively [16]. Edge thickness indicated association strength, with blue for positive associations and red for negative associations.

#### Network stability

We assessed edge and centrality stability in the three networks using bootnet package [16]. Edge weight stability was determined through nonparametric bootstrap, with 95% confidence intervals (CIs) indicating accuracy. A narrower CI represents a network of higher credibility [16]. Centrality stability was estimated using case-dropping subset bootstrap, measured by the Correlation Stability Coefficient (CS-C). CS-C value above 0.25, preferably surpassing 0.5, indicates optimal stability [16].

#### Centrality, bridge node and bridge centrality

The centrality of nodes in networks was determined using strength or expected influence indices [14]. In the presence of negative edges, the most central node was identified based on the highest expected influence index, which combined positive and negative edge values within the network. Likewise, the most important bridge node was determined by the highest bridge expected influence (one-step) index, which considered the sum of positive and negative edge values connecting a node to all nodes outside its community [18]. To determine whether a centrality index was significantly higher, we conducted a centrality bootstrapped difference test, with significance defined as non-containment of the corresponding 1000-bootstrap 95% non-parametric CI by zero [16]. We computed centrality indices using the qgraph package in R and bridge centrality indices using the bridge function of the networktools package. The bootnet package was used for the centrality bootstrapped difference test.

#### Network comparison test

To compare the three networks based on gender and occupation (health care vs. non-health care professionals), we used the NetworkComparisonTest package in R. We conducted a network invariance test and global strength invariance test. The former assessed significant differences in edges between subgroup networks, while the latter compared the weighted absolute sum of all edges, serving as a comparison of the intensity of connections among variables within networks. If the network invariance test was significant, an edge invariance test was then performed to identify specific pairs of edges that differed between subgroups. Node centrality between subgroups was also compared. To correct for multiple comparisons at the level of individual edges and centralities, we employed the Holm-Bonferroni correction method.

## RESULTS

### Sample characteristics and descriptions of lifestyles and interim health outcomes

**Figure 1:**
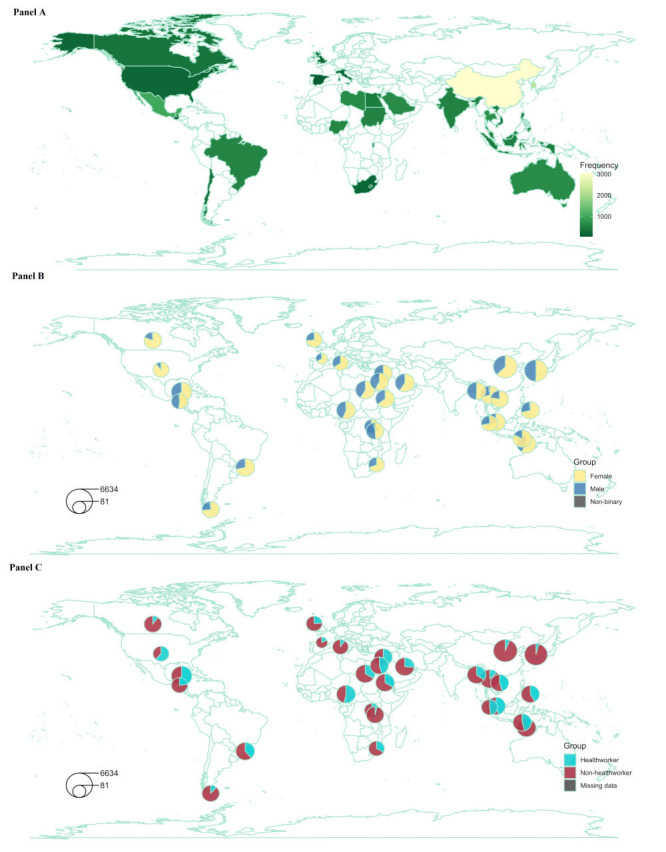
**Panel A.** Geographical distribution of overall sample. **Panel B.** Breakdown by gender. **Panel C.** Breakdown by occupation.

Out of 19 145 received responses, 16 512 were eligible for analysis (exclusions: blank/incomplete = 1940; duplicates = 116; inconsistent = 450; non-participating countries = 126; missing data = 1). Eligible participants included 25.1% health workers (n = 4145), with 62.7% females (n = 10 351), 36.7% males (n = 6061), and 0.6% non-binary (n = 100). Detailed sociodemographics are in **Table 1** and **Figure 1****.** **Figure 1**, panel A shows geographical distribution of participants, that are categorised by gender (**Figure 1**, panel B) and occupation (**Figure 1**, panel C). **Table 1** also lists lifestyle and interim health outcome items, their abbreviations, and means and standard deviations (SDs).

**Table 1:** Demographics of 16 512 respondents and descriptive statistics of measurement items

Variables	Mean (n)	SD (%)
**Demographics**		
Age, years		
*18-24*	4857	29.4%
*25-29*	2345	14.2%
*30-34*	1931	11.7%
*35-39*	1855	11.2%
*40-44*	1427	8.6%
*45-49*	1157	7.0%
*50-54*	975	5.9%
*55-59*	667	4.0%
*60-64*	699	4.2%
*> = 65*	599	3.6%
Country		
*Australia*	639	3.9%
*Brazil*	553	3.3%
*Burundi*	369	2.2%
*Canada*	368	2.2%
*Chile*	342	2.1%
*Egypt*	461	2.8%
*Guatemala*	229	1.4%
*Hong Kong*	2127	12.9%
*India*	529	3.2%
*Indonesia*	482	2.9%
*Italy*	203	1.2%
*Lebanon*	440	2.7%
*Libya*	645	3.9%
*Macau*	250	1.5%
*Mainland China*	667	4.0%
*Malaysia*	535	3.2%
*Mexico*	1016	6.2%
*Nigeria*	590	3.6%
*Philippines*	457	2.8%
*Republic of the Sudan*	538	3.3%
*Rwanda*	150	0.9%
*Saudi Arabia*	631	3.8%
*Singapore*	237	1.4%
*South Africa*	198	1.2%
*South Korea*	2238	13.6%
*Spain*	51	0.3%
*Thailand*	723	4.4%
*United Kingdom*	212	1.3%
*United States*	213	1.3%
*Vietnam*	419	2.5%
Marital status		
*Married/cohabitation/common-law*	7275	44.1%
*Single*	8504	51.5%
*Separated/divorced/widowed*	732	4.4%
*Missing data*	1	0.0%
Education		
*Primary or below*	405	2.5%
*Secondary*	2627	15.9%
*Associate degree*	1576	9.5%
*Bachelor*	6500	39.4%
*College*	2258	13.7%
*Graduate*	2974	18.0%
*Missing*	172	1.0%
Employment		
*Job seeking*	885	5.4%
*Laid off*	170	1.0%
*Not in workforce*	990	6.0%
*Retired*	614	3.7%
*Self-employed*	1309	7.9%
*Student*	4589	27.8%
*Working (> = 40 h/week)*	5196	31.5%
*Working (1-39 h/week)*	2759	16.71%
Lifestyles and interim health outcomes*		
*Food types in daily meals (L1)*	3.01	0.87
*Consumption of fruits and vegetables (L2)*	3.15	0.90
*Less consumption of frozen food/food products (L3)*	2.99	0.97
*Less consumption of snacks (L4)*	3.04	1.00
*Less soft drinks/juices/other sugary drinks (L5)*	3.20	1.03
*Having a meal at home (L6)*	3.86	0.99
*Cooking at home (L7)*	3.80	0.98
*Less eating takeout food (L8)*	3.05	1.19
*Taking alternative medicine or natural health products (L9)*	2.90	0.91
*Taking oral supplements/vitamins (L10)*	3.06	0.93
*Less smoking tobacco (L11)*	3.37	0.94
*Less alcohol consumption (L12)*	3.38	0.96
*Less duration of sitting (L13)*	2.35	0.98
*Less duration of screen time (L14)*	2.26	0.98
*Frequency of exercise (L15)*	2.81	1.08
*Duration of exercise (L16)*	2.78	1.07
*Type of exercise (L17)*	2.78	1.03
*Overall amount of exercise (L18)*	2.77	1.07
*Lose weight (H1)*	2.79	0.90
*Appetite (H2)*	3.13	0.83
*Physical health (H3)*	2.91	0.80
*Sleep quality (H4)*	2.86	0.96
*Quality of life (H5)*	2.71	0.98
*Less mental burden (H6)*	2.60	1.07
*Less emotional distress (H7)*	2.63	1.04
*Family disputes (H8)*	3.10	0.87
*Social support provided (H9)*	3.09	0.86
*Social support received (H10)*	2.97	0.84
*Social activities (H11)*	2.36	1.06
*Income (H12)*	2.65	0.93
*Less economic burden (H13)*	2.76	1.01

SD – standard deviation, h – hours

*Scored on a 5-point Likert scale: 1 = substantially reduced; 3 = no change; 5 = substantially increased.

### Items remained after checking for item redundancy

The Goldbricker analysis suggested the removal of one lifestyle from each of the following pairs: L17-L15, L16-L15 and L18-L17. Retaining L18, which offered a comprehensive assessment of exercise, meant L17 had to be removed. Additionally, one of L15 and L16 had to be removed, but removing either one in this round would have led to the removal of the other in a second-round re-run of the Goldbricker analysis due to redundancy with L18. Two rounds of analysis confirmed no further redundancy. Only L18 and L1-L14 remained as retained lifestyles. No further reduction was required for the 13 interim health outcomes. The final network models comprised 15 lifestyles and 13 interim health outcomes.

### Stability of three networks

All three networks (lifestyle, interim health outcome, and bridge) showed accurate estimates for the edge-weights. The bootstrapped 95% CI analysis indicated precise edge-weight estimates with narrow CIs (Figures S1, S3 and S5 in the **Online Supplementary Document**). Additionally, the CS-C values of expected influence or bridge expected influence were all 0.75 (Figures S2, S4 and S6 in the **Online Supplementary Document**), surpassing the recommended threshold of 0.5 and demonstrating the interpretability of the three networks.

### Network of lifestyles

**Figure 2:**
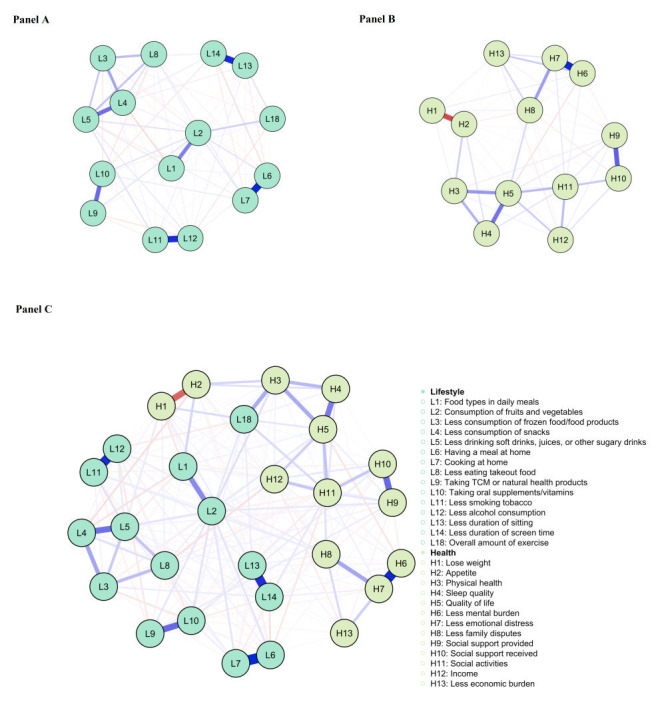
**Panel A.** Network structure of lifestyles. **Panel B.** Network structure of interim health outcomes. **Panel C.** Bridge network structure combining lifestyles and interim health outcomes.

**Figure 3:**
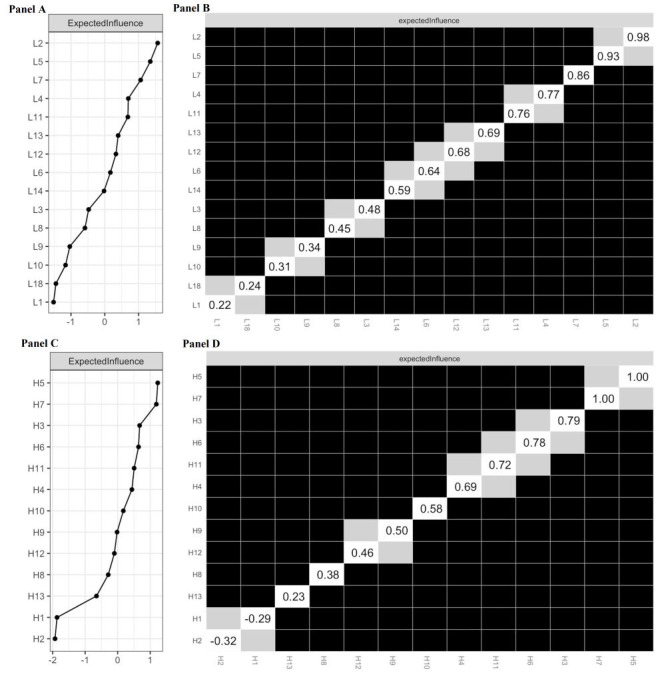
**Panel A.** Expected influence centrality index for variables in the lifestyle network. **Panel B.** Centrality bootstrapped difference tests for variables in the lifestyle network. **Panel C.** Expected influence centrality index for variables in the interim health outcomes network. **Panel D.** Centrality bootstrapped difference tests for variables in the interim health outcomes network. A grey cell indicates no significant difference between the corresponding two variables. A dark cell indicates significant difference between the corresponding two variables at 5% level of significance. A white cell shows the value of expected influence.

The network structure is depicted in **Figure 2**, panel A. Of the 105 edges, 94 (89.5%) were estimated to be nonzero, indicating close connectivity between the nodes. The three largest edges were L6-L7 (0.68), L13-L14 (0.62) and L11-L12 (0.61). Table S1 in the **Online Supplementary Document** presents the partial correlation matrix for other edges. Additionally, **Figure 3**, panel A displays the expected influence index for all nodes. L2 had the highest expected influence among the 15 lifestyles. However, the centrality bootstrapped difference test indicated no significant difference between L2 and L5. Both L2 and L5 had significantly higher expected influence than other variables (**Figure 3**, panel B), making them equally influential in activating or deactivating other nodes for an overall healthier lifestyle.

### Network of interim health outcomes

**Figure 2**, panel B illustrates the network structure. Most edges (67/78, 85.9%) showed nonzero values, indicating close connectivity between nodes. The three largest edges were H6 and H7 (0.65), H1 and H2 (-0.46), and H9 and H10 (0.41). Table S2 in the **Online Supplementary Document** presents the partial correlation matrix for the remaining edges. Additionally, **Figure 3**, panel C displays the expected influence index for all nodes. H5 had the highest expected influence among the 13 interim health outcomes. The centrality bootstrapped difference test indicated no significant difference between H5 and H7 (**Figure 3**, panel D) and both were significantly higher than all other interim health outcomes. Thus, H5 and H7 jointly served as the most important nodes with strong influences on other nodes.

### Bridge network of lifestyles and interim health outcomes

**Figure 4:**
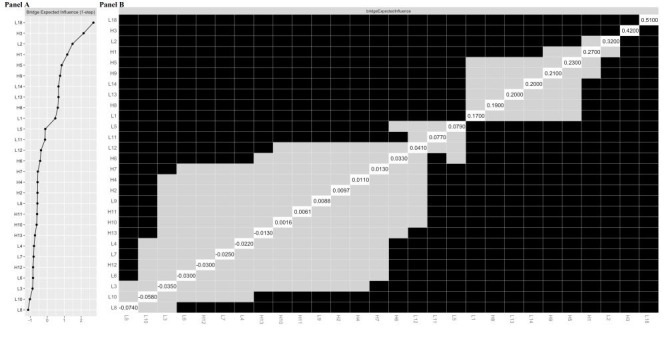
**Panel A.** Bridge expected influence centrality index for variables in the bridge network. **Panel B.** Centrality bootstrapped difference tests for variables in the bridge network. A grey cell indicates no significant difference between the corresponding two variables. A dark cell indicates significant difference between the corresponding two variables at 5% level of significance. A white cell shows the value of expected influence.

**Figure 2**, panel C illustrates the network structure. Among the 378 edges, 263 (69.6%) were nonzero, indicating strong node connectivity. Table S3 in the **Online Supplementary Document** provides additional edge details. Additionally, **Figure 4**, panel A displays the bridge expected influence index for all nodes within the network. L18 exhibited the highest bridge expected influence among the 15 lifestyles, followed by L2 and L14. The corresponding bridge edges were L18-H3, L2-H3, and L14-H11. The centrality bootstrapped difference test confirmed that L18 significantly surpassed all other nodes (**Figure 4**, panel B), indicating its greatest ability to influence all interim health outcome nodes and promote overall health.

### Gender and occupation differences in networks

No significant differences were found in global strength invariance tests across all three networks for males (n = 5762) and females (n = 9794), as well as non-health workers (n = 11 777) and health workers (n = 3875) (male vs. female: lifestyle: test statistic for global strength invariance (S) = 0.23, *P* = 0.327; interim health outcomes: S = 0.02, *P* = 0.951; bridge: S = 0.14, *P* = 0.875; health worker vs. non-health worker: lifestyle: S = 0.15, *P* = 0.727; interim health outcomes: S = 0.20, *P* = 0.723; bridge: S = 0.85, *P* = 0.751). However, significant differences were observed in network invariance tests for both gender and occupation subgroups (male vs. female: lifestyle: test statistic for network invariance (M) = 0.16, *P* = 0.001; interim health outcomes: M = 0.11, *P* = 0.001; bridge: M = 0.16, *P* = 0.001; health worker vs. non-health worker: lifestyle: M = 0.10, *P* = 0.001; interim health outcomes: M = 0.09, *P* = 0.004; bridge: M = 0.09, *P* = 0.002). Specific edges that differed between subgroups in each network are listed in Table S4 in the **Online Supplementary Document**. Centrality invariance tests showed no significant differences in expected influence of the central variables or bridge expected influence of the bridge lifestyle between gender or occupation subgroups in all three networks (all *P* > 0.05). Supplementary Tables S5 and S6 in the **Online Supplementary Document** provide centrality comparisons of each variable between subgroups within each network.

## DISCUSSION

This study utilised a large and diverse international sample to unveil the complex interplay between lifestyles and interim health outcomes, while also yielding three significant additional findings that shed light on key variables for interventions. First, consuming fruits and vegetables along with drinking less sugary drinks were tied for the most central lifestyles among the 15 examined, while quality of life together with less emotional distress were jointly the most central interim health outcomes among the 13 studied. Modifying them can lead to substantial overall changes in their groups. Second, among all 15 lifestyles examined, the overall amount of exercise (bridge lifestyle) demonstrated the strongest association with all 13 interim health outcomes, indicating its substantial influence on overall health when modified. Considering the well-established cause-and-effect relationship between lifestyle and health, increasing the overall exercise amount could bring significant improvements in overall health. Lastly, no significant differences were observed based on global strength invariance and centrality of central or bridge nodes in the network when comparing gender and occupation (health worker and non-health worker), suggesting that tailored interventions for specific subgroups may not be necessary.

The centrality of fruits and vegetables and sugary drinks among the 15 lifestyles examined can be attributed to their associations with other healthy or harmful behaviours. Prioritising the consumption of fruits and vegetables in one's diet is associated with adopting other healthy behaviours, such as physical activity, and avoiding harmful practices like smoking and excessive alcohol consumption [19]. Sugary drinks can increase energy intake and fat storage, trigger a dopamine release that can be addictive, and be associated with physical inactivity and other addictive behaviours [20,21]. Recent findings also associate sugary drinks with increased all-cause cancer risk [22], indirectly supporting their central role in overall lifestyles. Additionally, quality of life emerges as the central variable among the 13 interim health outcomes, given its multidimensional nature, encompassing physical health, mental health and social relationships [23]. Similarly, emotional distress holds equal centrality among interim health outcomes due to its profound impact on both physical and mental well-being, including elevated levels of stress hormones, weakened immune function, heightened risk of chronic conditions, disrupted sleep and diet patterns, poorer physical health, and mental health conditions such as depression and anxiety [24]. These central variables play a pivotal role in shaping an individual's overall healthy lifestyle or achieving better overall interim health outcomes. While our study focused on a limited number of interim health outcomes, interventions targeting the central lifestyle variable could lead to significant overall improvements across all aspects of lifestyle and hold the potential to improve other difficult-to-measure or low prevalence lifestyle-related health outcomes among the general population, such as mortality, cardiovascular disease risk and severe mental illness. Furthermore, interventions targeting the central interim health outcome variable can directly improve general interim health outcomes, particularly when seeking to improve health through avenues other than lifestyle modifications. For example, implementing interventions such as promoting education, improving access to health care, and implementing economic policies that enhance quality of life and emotional well-being could lead to significant improvements in overall interim health outcomes.

The significance of the overall amount of exercise as the most influential lifestyle factor on overall interim health outcomes highlights its paramount importance as a target for interventions or improvements. Its pivotal role can be attributed to the well-established relationship between exercise and various mental and physical well-being indicators [25]. Regular physical activity not only promotes individual well-being but also benefits people of all ages and abilities. Our study provides novel evidence of the irreplaceable and critical role of exercise among all modifiable lifestyles in enhancing overall health. Thus, it should be a primary and fundamental objective of both health interventions and public health policies, demanding substantial resources and concerted efforts to achieve a wide-ranging impact and maximise overall well-being. It is worth noting that the COVID-19 pandemic has exacerbated existing inequities in access and opportunities for being physically active, leading to a more sedentary lifestyle for many individuals. In the face of declining physical activity levels, policies should exert significant efforts to encourage and promote physical activity, mitigating the detrimental effect on overall health.

The absence of significant differences in the strength of connections and centrality of key variables within each network between gender and occupation (health worker and non-health worker) suggests that the interplay between lifestyles and interim health outcomes may operate through a uniform mechanism across these demographic groups. In the realm of health care, this finding suggests that interventions targeting key variables can yield similar effects regardless of gender or occupation, obviating the need for tailored interventions. However, subtle distinctions were observed at specific edges and non-central nodes among subgroups, emphasising the importance of considering gender and occupation differences when designing health interventions or formulating policies aimed at promoting specific lifestyles and interim health outcomes that show variations. Future studies should explore these differences to better understand the underlying mechanisms and develop more effective interventions for specific subgroups.

When shifting from a holistic or systems-level perspective to a more nuanced examination of specific paired variables, we can glean practical implications for health promotion practice by focusing on the strongest associations within each network. Notably, the top three associations within both the lifestyle and interim health outcome networks have already been extensively documented in previous studies [26-30]. However, this study validates that the magnitude of their association is the strongest among their groups, with the added robustness of network analysis that accounts for all others as confounding variables. Applications of these findings include optimising resource allocation through the integration of smoking and alcohol cessation interventions, highlighting the role of appetite in weight management and emphasising the need for future interventions to consider the reciprocal effects of these variables for added efficacy.

### Limitations

Our study has several limitations. First, our online convenience sampling method might have resulted in the underrepresentation of individuals with low socio-economic status or limited digital access, while potentially overrepresenting health care workers. However, network comparison tests showed no significant differences in the primary outcomes between health care workers and other respondents, indicating that the overrepresentation of health care workers had minimal influence on our findings. Second, we relied on self-reported data, which calls for cautious interpretation due to potential reliability concerns. Third, the cross-sectional nature of our study limits our ability to establish causality or capture the sequential dynamics between lifestyle factors and interim health outcomes, highlighting the importance of longitudinal studies. Fourth, we recognise that our study primarily focused on quantifiable health-related behaviours, potentially overlooking the underlying beliefs and experiences that shape them. Consequently, interventions based on our findings should address both the observable behaviours and the underlying beliefs driving them. Fifth, our study primarily focused on lifestyle, overlooking other influential factors of health such as socioeconomics, physical environment, personal health practices, individual capacity, coping skills, and health services. Future studies should adopt a more holistic perspective to capture the complex interplay of these determinants of health. Sixth, our findings may not be generalisable to other large-scale emergency events, such as world wars or major flu outbreaks, as their effects on lifestyles and interim health outcomes may differ from the COVID-19 pandemic. Lastly, the approach of assessing changes in outcomes relative to pre-pandemic conditions rather than the current status may limit the applicability of the findings to the post-pandemic period. However, assuming a rebound effect with equal magnitude but opposite direction on the assessed outcomes could make the findings relevant post-pandemic. Nevertheless, future studies conducted during non-pandemic periods are necessary to further validate these findings.

## CONCLUSIONS

The findings of this study provide valuable insights into the future of health care for general populations. The paramount significance of consuming fruits and vegetables while reducing the intake of sugary drinks has emerged as equally important lifestyle factors, holding the potential to enhance substantial improvements in a broader range of health outcomes beyond those included in this study. Improvements in quality of life and decreased emotional distress were the most central interim health outcomes, underscoring the transformative possibilities of interventions (extending beyond lifestyle modifications) targeting these factors in effecting significant changes in overall interim health outcomes. Furthermore, the overall amount of exercise, a bridge lifestyle, has the strongest connection with overall interim health outcomes, suggesting its indispensability for the general population in improving their interim health outcomes through lifestyle interventions. Moreover, strong associations between specific lifestyle factors, such as smoking and alcohol consumption, as well as appetite and weight loss further highlight the prospects of integrated interventions that can improve cost-effectiveness.

## Additional material


Online Supplementary Document
