# Supplementary material for: Key lifestyles and interim health outcomes for effective interventions in general populations: A network analysis of a large international observational study
Source: J Glob Health. 2023 Oct 20;13:04125. doi: 10.7189/jogh.13.04125 (PMC10588292; doi:10.7189/jogh.13.04125)
Supplement: Online Supplementary Document [file jogh-13-04125-s001.pdf]

ONLINE SUPPLEMENTARY DOCUMENT

Title: Key lifestyles and interim health outcomes for effective interventions in general populations: A network analysis of a large international observational study

Authors: Jiaying Li, Daniel Yee Tak Fong, Kris Yuet Wan Lok, Janet Yuen Ha Wong, Mandy Man Ho, Edmond Pui Hang Choi, Vinciya Pandian, Patricia M Davidson, Wenjie Duan, Marie Tarrant, Jung Jae Lee, Chia-Chin Lin, Oluwadamilare Akingbade, Khalid M Alabdulwahhab, Mohammad Shakil Ahmad, Mohamed Alboraie, Meshari A Alzahrani, Anil S Bilimale, Sawitree Boonpatcharanon, Samuel Byiringiro, Muhammad Kamil Che Hasan, Luisa Clausi Schettini, Walter Corzo, Josephine M. De Leon, Anjanette S. De Leon, Hiba Deek, Fabio Efficace, Mayssah A El Nayal, Fathiya El-Raey, Eduardo Ensaldo-Carrasco, Pilar Escotorin, Oluwadamilola Agnes Fadodun, Israel Opeyemi Fawole, Yong-Shian Shawn Goh, Devi Irawan, Naimah Ebrahim Khan, Binu Koirala, Ashish Krishna, Cannas Kwok, Tung Thanh Le, Daniela Giambruno Leal, Miguel Ángel Lezana-Fernández, Emery Manirambona, Leandro Cruz Mantoani, Fernando Meneses-González, Iman Elmahdi Mohamed, Madeleine Mukeshimana, Chinh Thi Minh Nguyen, Huong Thi Thanh Nguyen, Khanh Thi Nguyen, Son Truong Nguyen, Mohd Said Nurumal, Aimable Nzabonimana, Nagla Abdelrahim Mohamed Ahmed Omer, Oluwabunmi Ogungbe, Angela Chiu Yin Poon, Areli Reséndiz-Rodriguez, Busayasachee Puang-Ngern, Ceryl G Sagun, Riyaz Ahmed Shaik, Nikhil Gauri Shankar, Kathrin Sommer, Edgardo Toro, Hanh Thi Hong Tran, Elvira L Urgel, Emmanuel Uwiringiyimana, Tita Vanichbuncha, Naglaa Youssef

Supplementary material summary

**Contents**	**Numbered pages**
Table S1. Partial correlation matrix of variables in lifestyles network	2
Table S2. Partial correlation matrix of variables in interim health outcomes network	3
Table S3. Partial correlation matrix of variables in bridge network of lifestyles and interim health outcomes	4-6
Table S4. Significant edges in network comparison test for all three networks.	7-8
Table S5. Centrality invariance test on expected influence for network of lifestyles and interim health outcomes.	9
Table S6. Bridge centrality invariance test on bridge expected influence for the bridge network models.	10
Figure S1. Bootstrapped confidence intervals of edge weights for the lifestyles network.	11
Figure S2. The stability of expected influence centrality index in lifestyles network using case-dropping bootstrap.	12
Figure S3. Bootstrapped confidence intervals of edge weights for the interim health outcomes network.	13
Figure S4. The stability of expected influence centrality index in interim health outcomes network using case-dropping bootstrap.	14

Figure S5. Bootstrapped confidence intervals of edge weights for the bridge network model.	15
Figure S6. The stability of bridge expected influence centrality index in bridge network using case-dropping bootstrap.	16

Table S1. Partial correlation matrix of variables in lifestyles network.

	L1	L2	L3	L4	L5	L6	L7	L8	L9	L10	L11	L12	L13	L14	L18
L1	-	0.3393	-0.0240	-0.0967	-0.0659	0.0337	0.0079	-0.0075	0.0276	0.0000	0.0136	-0.0100	-0.0147	0.0000	0.0181
L2	0.3393	-	-0.0480	0.0481	0.0784	0.0741	0.0671	0.0461	0.0722	0.0772	0.0443	0.0240	0.0210	0.0090	0.1292
L3	-0.0240	-0.0480	-	0.2417	0.1422	-0.0300	-0.0284	0.1786	-0.0058	-0.0601	0.0374	0.0006	0.0000	0.0440	0.0296
L4	-0.0967	0.0481	0.2417	-	0.3880	-0.0028	0.0000	0.0863	-0.0023	-0.0016	0.0000	0.0314	0.0423	0.0328	0.0000
L5	-0.0659	0.0784	0.1422	0.3880	-	0.0000	0.0429	0.1580	0.0000	0.0000	0.0803	0.0585	0.0000	0.0377	0.0078
L6	0.0337	0.0741	-0.0300	-0.0028	0.0000	-	0.6822	-0.0361	0.0086	0.0010	0.0140	0.0360	-0.0499	-0.0809	-0.0136
L7	0.0079	0.0671	-0.0284	0.0000	0.0429	0.6822	-	0.0536	0.0057	0.0546	0.0237	0.0031	-0.0293	-0.0508	0.0251
L8	-0.0075	0.0461	0.1786	0.0863	0.1580	-0.0361	0.0536	-	-0.0308	-0.0641	0.0274	0.0387	-0.0032	0.0178	-0.0140
L9	0.0276	0.0722	-0.0058	-0.0023	0.0000	0.0086	0.0057	-0.0308	-	0.4034	-0.0457	-0.0647	-0.0230	-0.0052	0.0000
L10	0.0000	0.0772	-0.0601	-0.0016	0.0000	0.0010	0.0546	-0.0641	0.4034	-	-0.0387	-0.0332	-0.0033	-0.0598	0.0335
L11	0.0136	0.0443	0.0374	0.0000	0.0803	0.0140	0.0237	0.0274	-0.0457	-0.0387	-	0.6084	0.0220	0.0000	-0.0227
L12	-0.0100	0.0240	0.0006	0.0314	0.0585	0.0360	0.0031	0.0387	-0.0647	-0.0332	0.6084	-	0.0406	0.0050	-0.0614
L13	-0.0147	0.0210	0.0000	0.0423	0.0000	-0.0499	-0.0293	-0.0032	-0.0230	-0.0033	0.0220	0.0406	-	0.6120	0.0787
L14	0.0000	0.0090	0.0440	0.0328	0.0377	-0.0809	-0.0508	0.0178	-0.0052	-0.0598	0.0000	0.0050	0.6120	-	0.0286
L18	0.0181	0.1292	0.0296	0.0000	0.0078	-0.0136	0.0251	-0.0140	0.0000	0.0335	-0.0227	-0.0614	0.0787	0.0286	-

Table S2. Partial correlation matrix of variables in interim health outcomes network.

	H1	H2	H3	H4	H5	H6	H7	H8	H9	H10	H11	H12	H13
H1	-	-0.4585	0.0381	-0.0140	0.0234	0.0456	0.0010	0.0393	0.0045	-0.0192	0.0086	0.0000	0.0370
H2	-0.4585	-	0.1256	0.0787	0.0015	-0.0156	-0.0229	-0.0495	0.0172	0.0000	-0.0071	0.0275	-0.0171
H3	0.0381	0.1256	-	0.2010	0.2649	0.0000	0.0000	0.0134	0.0471	0.0160	0.0405	0.0396	0.0088
H4	-0.0140	0.0787	0.2010	-	0.3564	0.0000	0.0223	-0.0286	-0.0060	0.0117	0.0681	0.0040	0.0000
H5	0.0234	0.0015	0.2649	0.3564	-	-0.0710	0.0000	0.0997	0.0528	0.0000	0.1768	0.1298	0.0000
H6	0.0456	-0.0156	0.0000	0.0000	-0.0710	-	0.6525	0.0258	-0.0426	0.0124	0.0442	-0.0004	0.1301
H7	0.0010	-0.0229	0.0000	0.0223	0.0000	0.6525	-	0.2511	-0.0205	0.0000	0.0585	0.0000	0.0740
H8	0.0393	-0.0495	0.0134	-0.0286	0.0997	0.0258	0.2511	-	-0.0307	-0.0524	-0.0206	0.0275	0.1067
H9	0.0045	0.0172	0.0471	-0.0060	0.0528	-0.0426	-0.0205	-0.0307	-	0.4077	0.0605	0.0427	-0.0348
H10	-0.0192	0.0000	0.0160	0.0117	0.0000	0.0124	0.0000	-0.0524	0.4077	-	0.1436	0.0718	-0.0112
H11	0.0086	-0.0071	0.0405	0.0681	0.1768	0.0442	0.0585	-0.0206	0.0605	0.1436	-	0.1686	-0.0184
H12	0.0000	0.0275	0.0396	0.0040	0.1298	-0.0004	0.0000	0.0275	0.0427	0.0718	0.1686	-	-0.0487
H13	0.0370	-0.0171	0.0088	0.0000	0.0000	0.1301	0.0740	0.1067	-0.0348	-0.0112	-0.0184	-0.0487	-

Table S3. Partial correlation matrix of variables in bridge network of lifestyles and interim health outcomes.

	**L1**	**L2**	**L3**	**L4**	**L5**	**L6**	**L7**	**L8**	**L9**	**L10**	**L11**	**L12**	**L13**	**L14**	**L18**
L1	0.0000	0.3098	-0.0257	-0.0735	-0.0513	0.0299	0.0039	0.0000	0.0250	0.0000	0.0026	0.0000	0.0000	0.0000	0.0000
L2	0.3098	0.0000	-0.0406	0.0383	0.0700	0.0710	0.0692	0.0433	0.0666	0.0747	0.0383	0.0187	0.0055	0.0010	0.0622
L3	-0.0257	-0.0406	0.0000	0.2385	0.1408	-0.0333	-0.0221	0.1721	-0.0059	-0.0599	0.0338	0.0005	0.0000	0.0494	0.0164
L4	-0.0735	0.0383	0.2385	0.0000	0.3784	0.0000	0.0000	0.0825	-0.0004	-0.0003	0.0000	0.0281	0.0324	0.0234	0.0000
L5	-0.0513	0.0700	0.1408	0.3784	0.0000	0.0000	0.0383	0.1528	0.0000	0.0000	0.0777	0.0563	0.0000	0.0305	0.0011
L6	0.0299	0.0710	-0.0333	0.0000	0.0000	0.0000	0.6737	-0.0266	0.0063	0.0000	0.0131	0.0343	-0.0439	-0.0730	0.0000
L7	0.0039	0.0692	-0.0221	0.0000	0.0383	0.6737	0.0000	0.0400	0.0038	0.0489	0.0244	0.0026	-0.0238	-0.0440	0.0174
L8	0.0000	0.0433	0.1721	0.0825	0.1528	-0.0266	0.0400	0.0000	-0.0294	-0.0607	0.0265	0.0358	0.0000	0.0190	-0.0027
L9	0.0250	0.0666	-0.0059	-0.0004	0.0000	0.0063	0.0038	-0.0294	0.0000	0.3953	-0.0403	-0.0607	-0.0172	-0.0014	0.0000
L10	0.0000	0.0747	-0.0599	-0.0003	0.0000	0.0000	0.0489	-0.0607	0.3953	0.0000	-0.0335	-0.0296	0.0000	-0.0476	0.0376
L11	0.0026	0.0383	0.0338	0.0000	0.0777	0.0131	0.0244	0.0265	-0.0403	-0.0335	0.0000	0.5990	0.0132	0.0000	-0.0248
L12	0.0000	0.0187	0.0005	0.0281	0.0563	0.0343	0.0026	0.0358	-0.0607	-0.0296	0.5990	0.0000	0.0324	0.0000	-0.0556
L13	0.0000	0.0055	0.0000	0.0324	0.0000	-0.0439	-0.0238	0.0000	-0.0172	0.0000	0.0132	0.0324	0.0000	0.5777	0.0500
L14	0.0000	0.0010	0.0494	0.0234	0.0305	-0.0730	-0.0440	0.0190	-0.0014	-0.0476	0.0000	0.0000	0.5777	0.0000	0.0105
L18	0.0000	0.0622	0.0164	0.0000	0.0011	0.0000	0.0174	-0.0027	0.0000	0.0376	-0.0248	-0.0556	0.0500	0.0105	0.0000
H1	-0.0650	0.0322	0.0065	0.0285	0.0561	-0.0083	-0.0034	0.0357	0.0000	-0.0035	0.0000	0.0108	0.0484	0.0449	0.0919
H2	0.1081	0.0000	0.0000	-0.0798	0.0000	0.0179	0.0142	0.0000	0.0000	0.0000	0.0098	-0.0056	-0.0286	-0.0263	0.0000
H3	0.0278	0.0834	0.0000	0.0161	0.0062	0.0000	0.0127	0.0000	0.0000	0.0000	0.0196	0.0106	0.0173	0.0044	0.2221
H4	0.0000	0.0000	0.0000	0.0000	-0.0011	0.0050	0.0000	0.0000	0.0000	-0.0218	0.0000	0.0000	0.0000	-0.0090	0.0375
H5	0.0357	0.0593	0.0267	0.0077	0.0000	0.0000	0.0000	0.0179	-0.0187	-0.0071	0.0142	0.0000	0.0204	0.0029	0.0665
H6	-0.0051	0.0000	-0.0110	0.0000	0.0000	0.0000	-0.0016	-0.0020	0.0000	0.0000	0.0000	0.0000	0.0276	0.0255	0.0000
H7	0.0000	0.0004	-0.0182	0.0015	0.0000	0.0000	-0.0002	-0.0114	-0.0079	-0.0262	0.0000	0.0108	0.0214	0.0429	0.0000

H8	0.0028	0.0406	0.0000	0.0154	0.0232	0.0000	0.0000	0.0000	-0.0286	0.0000	0.0510	0.0479	0.0371	0.0224	-0.0212
H9	0.0136	0.0615	0.0000	0.0000	0.0314	0.0222	0.0000	0.0309	0.0512	0.0000	0.0038	0.0000	-0.0039	0.0000	0.0000
H10	0.0000	0.0056	-0.0312	0.0000	0.0000	0.0000	0.0269	-0.0402	0.0148	0.0493	-0.0340	-0.0045	0.0000	-0.0043	0.0192
H11	0.0132	0.0000	0.0000	-0.0108	-0.0391	-0.0601	-0.0288	-0.0193	-0.0020	-0.0453	0.0000	0.0023	0.0374	0.0612	0.0974
H12	0.0308	0.0358	-0.0073	0.0000	0.0000	0.0000	-0.0295	-0.0777	0.0000	0.0202	0.0000	-0.0315	0.0113	0.0181	0.0000
H13	0.0062	0.0026	0.0000	0.0000	0.0024	-0.0069	-0.0150	-0.0079	0.0000	-0.0233	0.0127	0.0000	0.0081	0.0137	-0.0055

Table S3. Partial correlation matrix of variables in bridge network of lifestyles and interim health outcomes. (continued)

	**H1**	**H2**	**H3**	**H4**	**H5**	**H6**	**H7**	**H8**	**H9**	**H10**	**H11**	**H12**	**H13**
L1	-0.0650	0.1081	0.0278	0.0000	0.0357	-0.0051	0.0000	0.0028	0.0136	0.0000	0.0132	0.0308	0.0062
L2	0.0322	0.0000	0.0834	0.0000	0.0593	0.0000	0.0004	0.0406	0.0615	0.0056	0.0000	0.0358	0.0026
L3	0.0065	0.0000	0.0000	0.0000	0.0267	-0.0110	-0.0182	0.0000	0.0000	-0.0312	0.0000	-0.0073	0.0000
L4	0.0285	-0.0798	0.0161	0.0000	0.0077	0.0000	0.0015	0.0154	0.0000	0.0000	-0.0108	0.0000	0.0000
L5	0.0561	0.0000	0.0062	-0.0011	0.0000	0.0000	0.0000	0.0232	0.0314	0.0000	-0.0391	0.0000	0.0024
L6	-0.0083	0.0179	0.0000	0.0050	0.0000	0.0000	0.0000	0.0000	0.0222	0.0000	-0.0601	0.0000	-0.0069
L7	-0.0034	0.0142	0.0127	0.0000	0.0000	-0.0016	-0.0002	0.0000	0.0000	0.0269	-0.0288	-0.0295	-0.0150
L8	0.0357	0.0000	0.0000	0.0000	0.0179	-0.0020	-0.0114	0.0000	0.0309	-0.0402	-0.0193	-0.0777	-0.0079
L9	0.0000	0.0000	0.0000	0.0000	-0.0187	0.0000	-0.0079	-0.0286	0.0512	0.0148	-0.0020	0.0000	0.0000
L10	-0.0035	0.0000	0.0000	-0.0218	-0.0071	0.0000	-0.0262	0.0000	0.0000	0.0493	-0.0453	0.0202	-0.0233
L11	0.0000	0.0098	0.0196	0.0000	0.0142	0.0000	0.0000	0.0510	0.0038	-0.0340	0.0000	0.0000	0.0127
L12	0.0108	-0.0056	0.0106	0.0000	0.0000	0.0000	0.0108	0.0479	0.0000	-0.0045	0.0023	-0.0315	0.0000
L13	0.0484	-0.0286	0.0173	0.0000	0.0204	0.0276	0.0214	0.0371	-0.0039	0.0000	0.0374	0.0113	0.0081
L14	0.0449	-0.0263	0.0044	-0.0090	0.0029	0.0255	0.0429	0.0224	0.0000	-0.0043	0.0612	0.0181	0.0137
L18	0.0919	0.0000	0.2221	0.0375	0.0665	0.0000	0.0000	-0.0212	0.0000	0.0192	0.0974	0.0000	-0.0055

H1	0.0000	-0.4055	0.0000	-0.0107	0.0000	0.0293	0.0000	0.0168	0.0000	-0.0036	0.0000	0.0000	0.0300
H2	-0.4055	0.0000	0.1099	0.0780	0.0000	-0.0128	-0.0101	-0.0366	0.0087	0.0000	0.0000	0.0184	-0.0114
H3	0.0000	0.1099	0.0000	0.1841	0.2256	0.0000	0.0000	0.0000	0.0295	0.0094	0.0200	0.0333	0.0030
H4	-0.0107	0.0780	0.1841	0.0000	0.3469	0.0000	0.0150	-0.0162	0.0000	0.0098	0.0651	0.0037	0.0000
H5	0.0000	0.0000	0.2256	0.3469	0.0000	-0.0663	0.0000	0.0772	0.0436	0.0000	0.1601	0.1281	0.0000
H6	0.0293	-0.0128	0.0000	0.0000	-0.0663	0.0000	0.6413	0.0209	-0.0388	0.0094	0.0281	0.0000	0.1271
H7	0.0000	-0.0101	0.0000	0.0150	0.0000	0.6413	0.0000	0.2361	-0.0145	0.0000	0.0401	0.0000	0.0692
H8	0.0168	-0.0366	0.0000	-0.0162	0.0772	0.0209	0.2361	0.0000	-0.0354	-0.0312	-0.0165	0.0255	0.0984
H9	0.0000	0.0087	0.0295	0.0000	0.0436	-0.0388	-0.0145	-0.0354	0.0000	0.3960	0.0686	0.0423	-0.0329
H10	-0.0036	0.0000	0.0094	0.0098	0.0000	0.0094	0.0000	-0.0312	0.3960	0.0000	0.1471	0.0602	-0.0024
H11	0.0000	0.0000	0.0200	0.0651	0.1601	0.0281	0.0401	-0.0165	0.0686	0.1471	0.0000	0.1511	-0.0237
H12	0.0000	0.0184	0.0333	0.0037	0.1281	0.0000	0.0000	0.0255	0.0423	0.0602	0.1511	0.0000	-0.0461
H13	0.0300	-0.0114	0.0030	0.0000	0.0000	0.1271	0.0692	0.0984	-0.0329	-0.0024	-0.0237	-0.0461	0.0000

Table S4. Significant edges in network comparison test for all three networks.

Significant edges	Network 1	Network 2	Absolute difference	*P value*
Lifestyles network (network 1: male vs network 2: female)				
L6 and L7	0.64	0.70	0.06	0.035
L8 and L9	-0.08	-0.01	0.07	0.035
L11 and L12	0.50	0.67	0.16	0.035
Lifestyles network (network 1: health worker vs network 2: non-health worker)				
L2 and L3	0.00	-0.07	0.07	0.026
L4 and L6	-0.03	0.00	0.03	0.030
L3 and L8	0.12	0.20	0.08	0.026
L8 and L9	-0.07	-0.01	0.06	0.030
L11 and L12	0..6587978	0.58	0.08	0.026
L6 and L14	-0.02	-0.12	0.10	0.026
L12 and L14	0.04	0.00	0.04	0.030
Interim health outcomes network (network 1: male vs network 2: female)				
H1 and H2	-0.39	-0.47	0.08	0.019
H6 and H7	0.58	0.69	0.11	0.019
H4 and H10	0.05	0.00	0.05	0.019
H9 and H10	0.36	0.44	0.08	0.019
H5 and H12	0.16	0.11	0.05	0.026
H10 and H12	0.10	0.04	0.06	0.026
Interim health outcomes network (network 1: health worker vs network 2: non-health worker)				
H1 and H2	-0.50	-0.42	0.07	0.019
H5 and H6	0.00	-0.08	0.08	0.019
H5 and H10	0.05	0.00	0.05	0.019
H3 and H12	0.00	0.06	0.06	0.039
H11 and H12	0.12	0.18	0.06	0.039
H12 and H13	-0.09	-0.01	0.09	0.019
Bridge network model (network 1: male vs network 2: female)				
L6 and L7	0.68	0.63	0.05	0.038
L8 and L9	-0.01	-0.08	0.07	0.038
L11 and L12	0.65	0.49	0.16	0.038
L6 and L13	-0.02	-0.08	0.06	0.038
H1 and H2	-0.42	-0.34	0.08	0.038
L2 and H7	0.00	0.02	0.02	0.038
H6 and H7	0.68	0.57	0.11	0.038
L12 and H8	0.02	0.09	0.07	0.038
H4 and H10	0.00	0.05	0.05	0.038
H9 and H10	0.42	0.35	0.07	0.038
Bridge network model (network 1: health worker vs network 2: non-health worker)				

L2 and L3	0.00	-0.06	0.06	0.025
L3 and L8	0.12	0.19	0.07	0.025
L8 and L9	-0.06	-0.01	0.05	0.025
L11 and L12	0.65	0.57	0.08	0.025
L6 and L14	-0.01	-0.11	0.09	0.025
L12 and L14	0.03	0.00	0.03	0.025
L13 and H1	0.00	0.07	0.07	0.040
H1 and H2	-0.45	-0.37	0.07	0.025
L7 and H3	0.04	0.00	0.04	0.040
L7 and H5	0.02	0.00	0.02	0.025
H5 and H6	0.00	-0.07	0.07	0.040
L8 and H7	0.01	-0.03	0.04	0.025
L9 and H7	-0.05	0.00	0.05	0.025
L10 and H7	-0.05	-0.01	0.04	0.025
L11 and H8	0.00	0.07	0.07	0.025
H5 and H10	0.04	0.00	0.04	0.025
L5 and H12	-0.04	0.00	0.04	0.025
H11 and H13	0.00	-0.04	0.04	0.040
H12 and H13	-0.08	0.00	0.08	0.025

Table S5. Centrality invariance test on expected influence for lifestyles network and interim health outcome network.

Variables	Expected influence		Expected influence	
	**Male vs female**	***p***	**Health worker vs non-health worker**	***p***
Lifestyles network model				
L1	-0.02	0.957	-0.07	0.524
L2	-0.08	0.463	0.01	1.000
L3	-0.17	0.030	0.02	1.000
L4	-0.05	0.470	-0.01	1.000
L5	-0.08	0.223	-0.05	0.710
L6	-0.11	0.082	0.18	0.060
L7	-0.13	0.060	0.13	0.157
L8	-0.08	0.223	-0.05	0.679
L9	-0.09	0.223	-0.13	0.157
L10	-0.05	0.604	0.09	0.414
L11	-0.04	0.693	0.05	0.710
L12	-0.20	0.030	0.11	0.157
L13	-0.06	0.470	0.00	1.000
L14	-0.03	0.803	0.12	0.157
L18	0.00	1.000	0.05	0.747
Interim health outcomes network model				
H1	0.12	0.121	-0.14	0.026
H2	0.07	0.364	-0.08	0.444
H3	0.11	0.364	-0.11	0.519
H4	-0.01	0.988	-0.07	0.444
H5	-0.01	0.988	0.08	0.519
H6	-0.04	0.957	-0.06	0.540
H7	-0.08	0.364	0.03	0.763
H8	0.03	0.988	-0.01	0.946
H9	-0.06	0.644	-0.09	0.405
H10	-0.04	0.988	0.24	0.017
H11	-0.05	0.753	0.00	1.000
H12	0.19	0.104	-0.25	0.017
H13	0.09	0.364	-0.17	0.017

Table S6. Bridge centrality invariance test on bridge expected influence for the bridge network models.

Variables	Bridge expected influence		Bridge expected influence	
	**Male vs female**	***p***	**Health worker vs non-health worker**	***p***
L1	-0.02	1.000	-0.02	0.825
L2	0.05	0.915	-0.12	0.157
L3	0.00	1.000	0.04	0.471
L4	0.01	1.000	0.01	0.960
L5	-0.02	1.000	-0.04	0.506
L6	-0.01	1.000	0.05	0.357
L7	-0.02	1.000	0.09	0.099
L8	-0.01	1.000	0.07	0.333
L9	0.05	0.700	-0.13	0.037
L10	0.08	0.476	-0.03	0.806
L11	-0.06	0.700	-0.07	0.232
L12	0.08	0.224	-0.06	0.278
L13	0.06	0.700	-0.17	0.037
L14	0.06	0.771	-0.02	0.807
L18	0.00	1.000	0.11	0.102
H1	-0.01	1.000	-0.02	0.852
H2	-0.05	0.700	0.06	0.351
H3	0.03	1.000	-0.03	0.806
H4	0.07	0.291	-0.03	0.466
H5	0.04	0.945	-0.02	0.806
H6	0.03	0.700	0.01	0.825
H7	0.05	0.700	-0.05	0.424
H8	0.01	1.000	-0.14	0.045
H9	0.02	1.000	-0.05	0.725
H10	-0.07	0.700	0.14	0.037
H11	0.01	1.000	-0.01	0.949
H12	0.05	0.779	-0.10	0.131
H13	0.07	0.476	-0.05	0.357

Figure S1. Bootstrapped confidence intervals of edge weights for the lifestyles network.

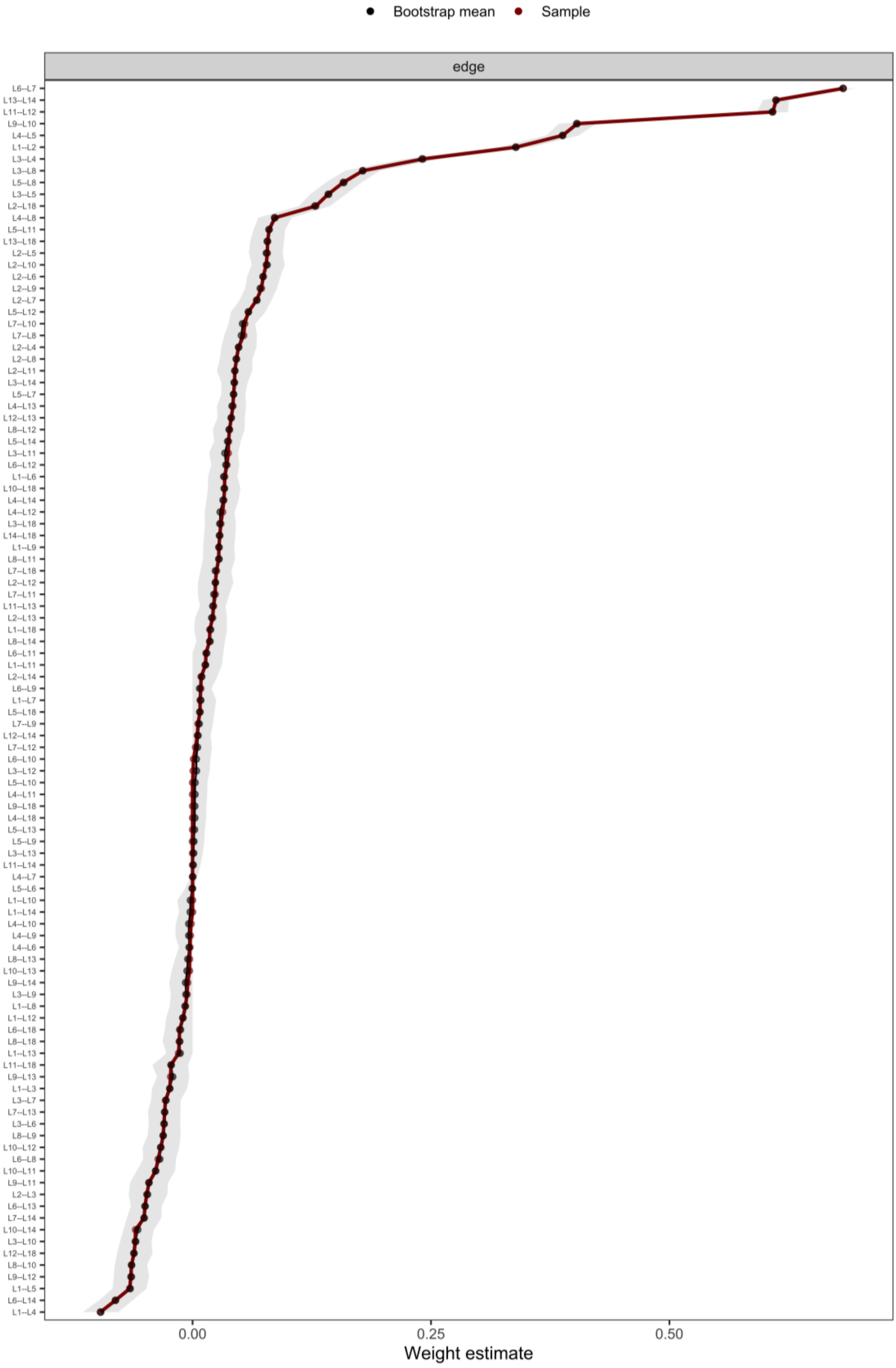

The red dots are sample means per edge, while the black dots are bootstrapped means, ordered from the highest to the lowest value. The gray area represents the 95% confidence intervals of edge weights, estimated with the non-parametric bootstrap procedure (Bootnet package). Wide intervals indicate lower stability and narrow intervals indicate higher stability.

Figure S2. The stability of expected influence centrality index in lifestyles network using case-dropping bootstrap.

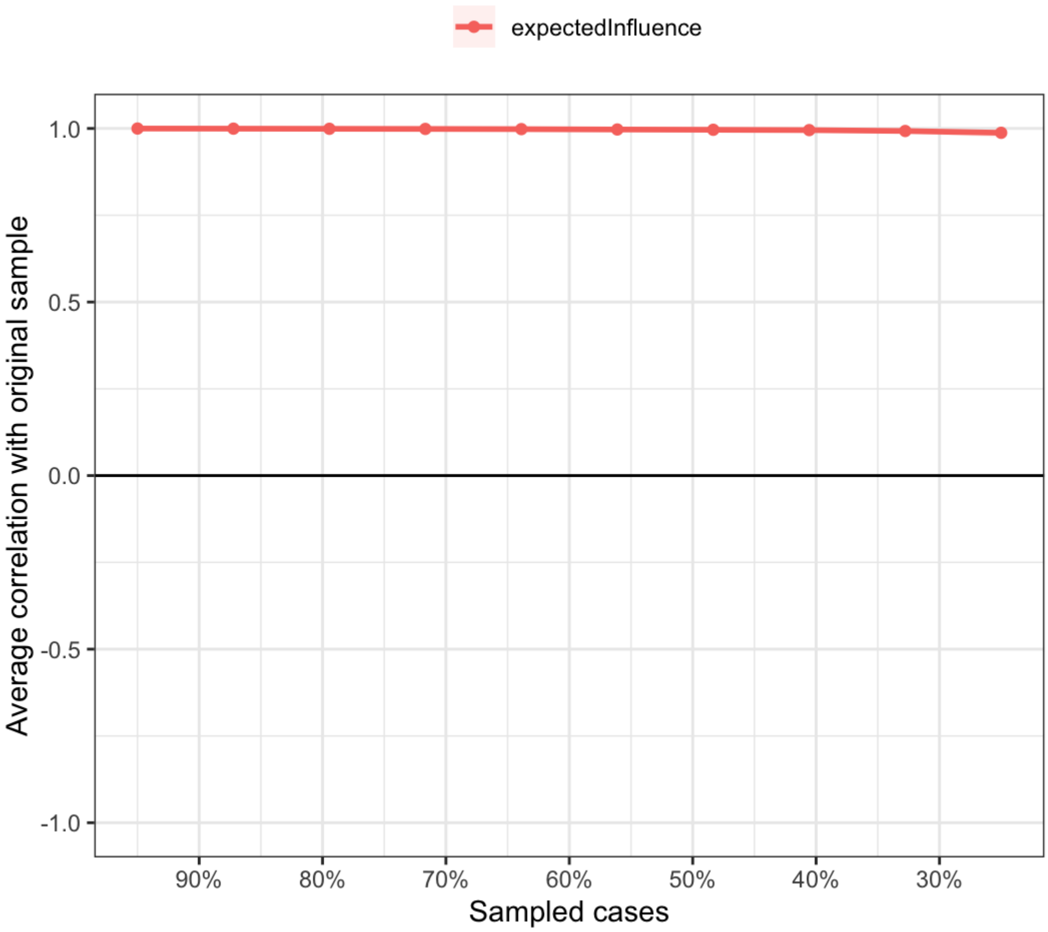

The x-axis indicates the percentage of cases of the original sample included at each step. The y-axis indicates the average of correlations between the expected influence centrality index from the original network and the expected influence centrality index from the networks that were re-estimated after excluding increasing percentages of cases.

Figure S3. Bootstrapped confidence intervals of edge weights for the interim health outcomes network.

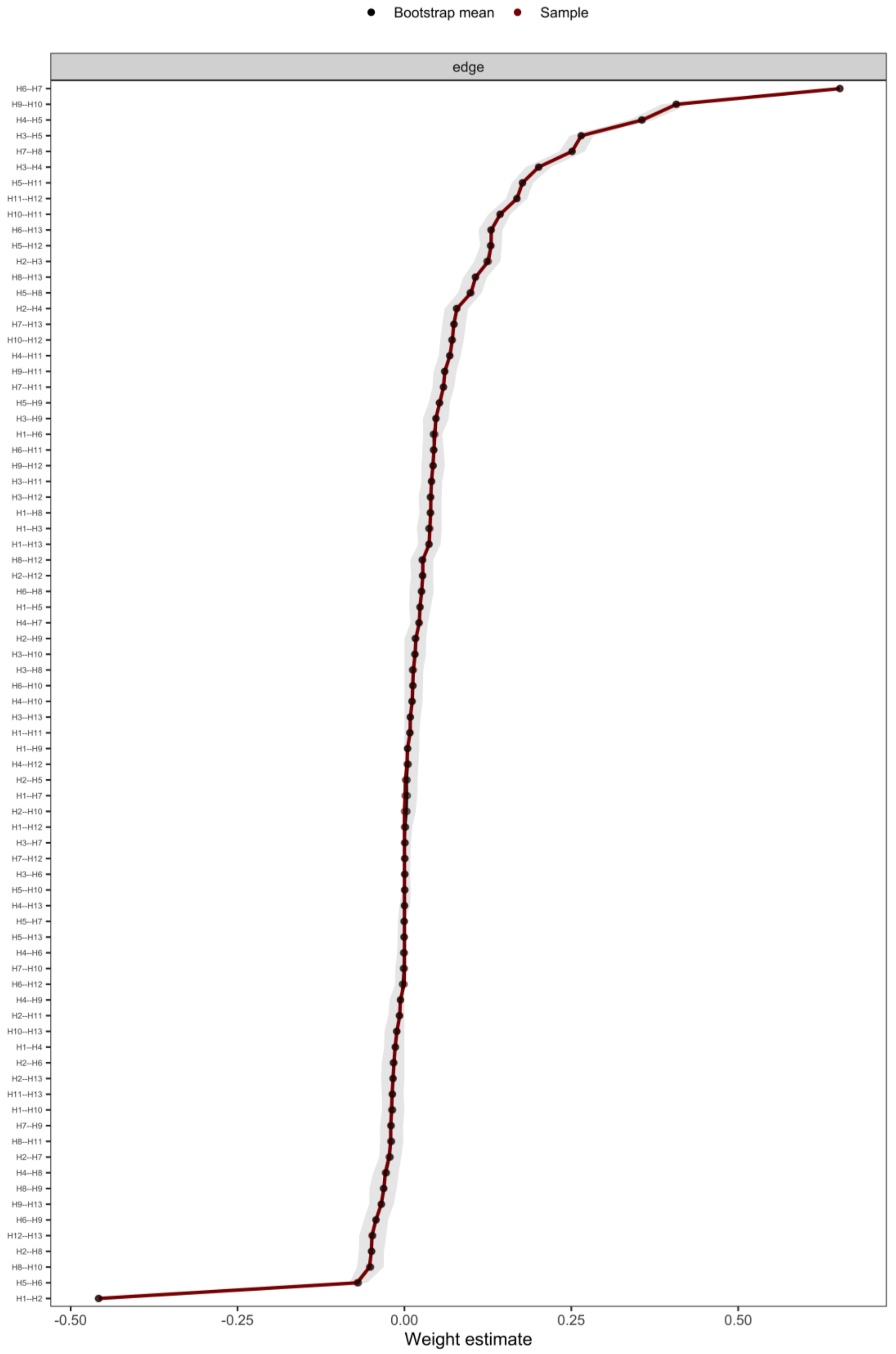

The red dots are sample means per edge, while the black dots are bootstrapped means, ordered from the highest to the lowest value. The gray area represents the 95% confidence intervals of edge weights, estimated with the non-parametric bootstrap procedure (Bootnet package). Wide intervals indicate lower stability and narrow intervals indicate higher stability.

Figure S4. The stability of expected influence centrality index in interim health outcomes network using case-dropping bootstrap.

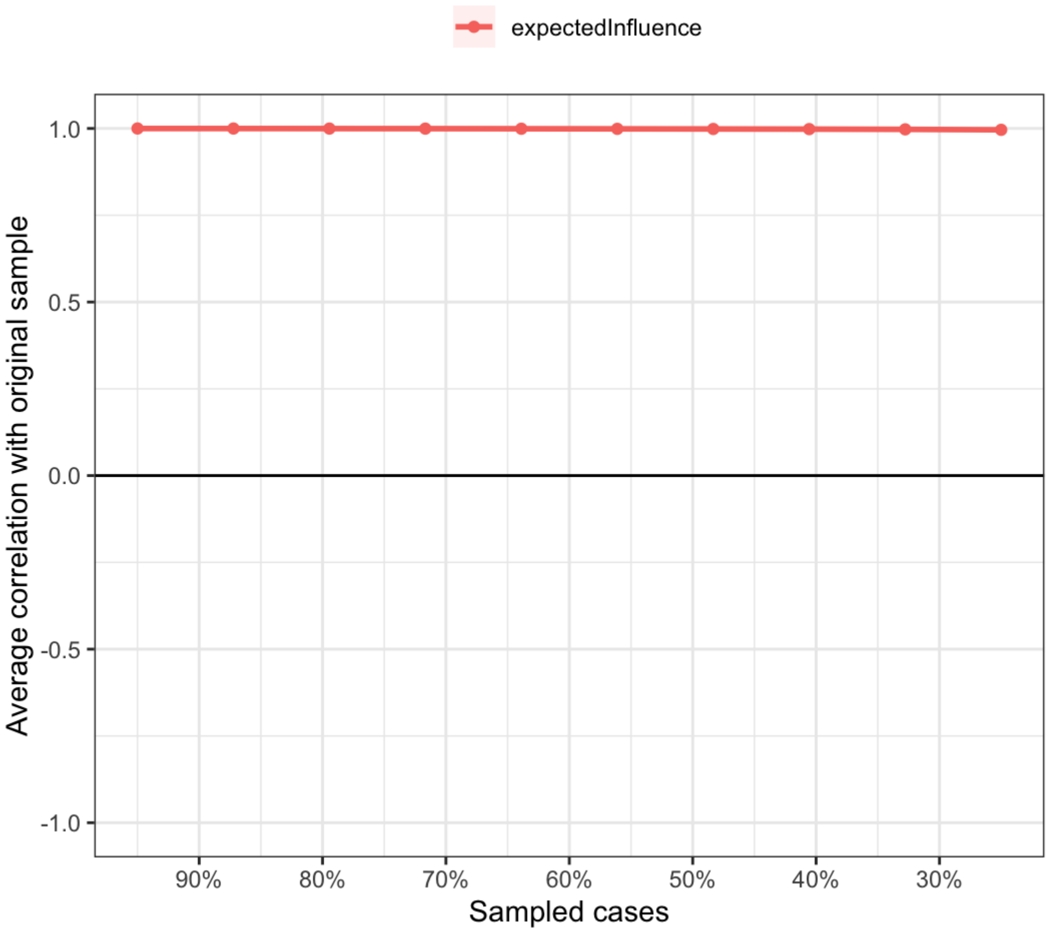

The x-axis indicates the percentage of cases of the original sample included at each step. The y-axis indicates the average of correlations between the expected influence centrality index from the original network and the expected influence centrality index from the networks that were re-estimated after excluding increasing percentages of cases.

Figure S5. Bootstrapped confidence intervals of edge weights for the bridge network.

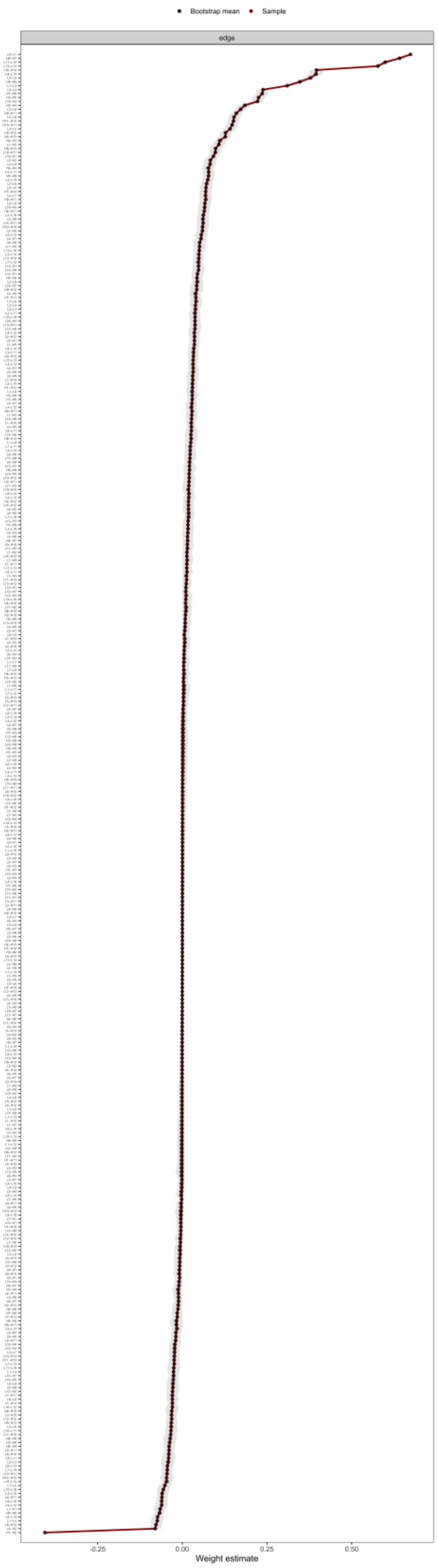

The red dots are sample means per edge, while the black dots are bootstrapped means, ordered from the highest to the lowest value. The gray area represents the 95% confidence intervals of edge weights, estimated with the non-parametric bootstrap procedure (Bootnet package). Wide intervals indicate lower stability and narrow intervals indicate higher stability.

Figure S6. The stability of bridge expected influence centrality index in bridge network using case-dropping bootstrap.

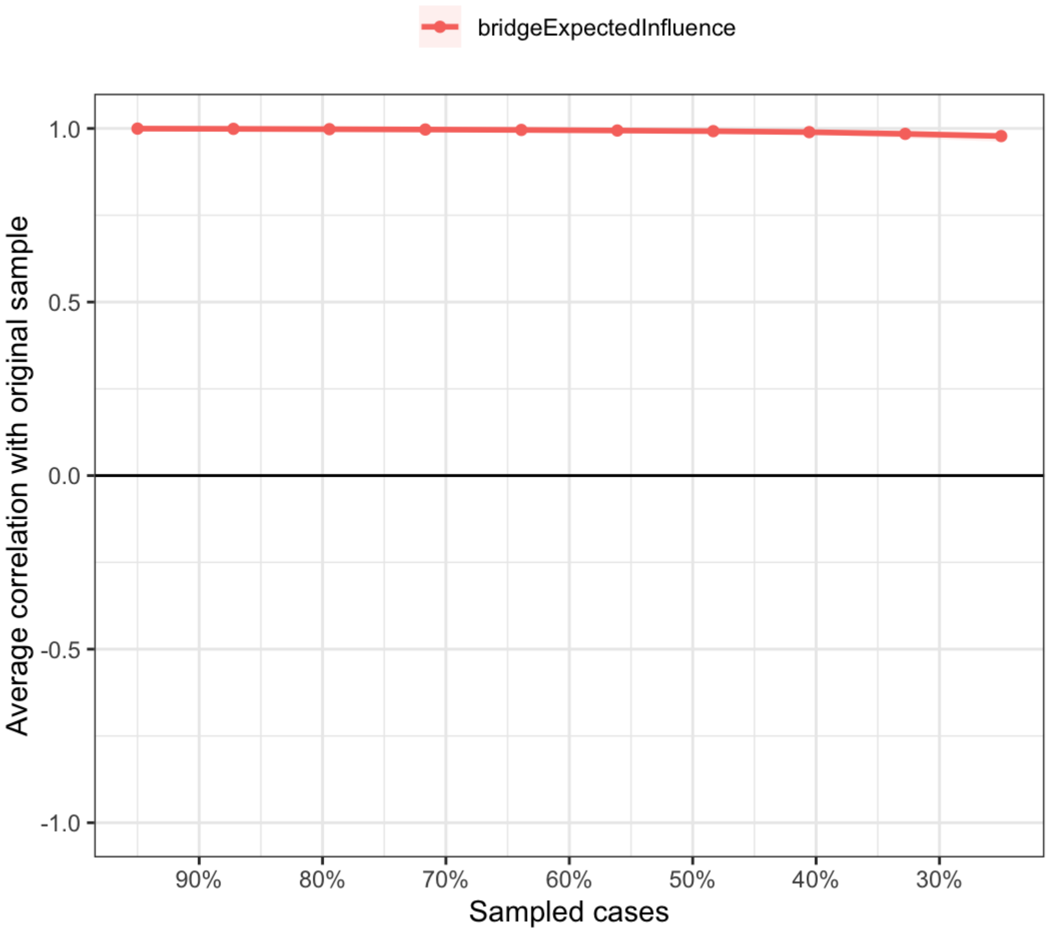

The x-axis indicates the percentage of cases of the original sample included at each step. The y-axis indicates the average of correlations between the bridge expected influence centrality index from the original network and the bridge expected influence centrality index from the networks that were re-estimated after excluding increasing percentages of cases.
